# Alcohol Use is Not Directly Related to the Perceived Control of Depressive Symptoms in Patients with Depressive Symptoms

**DOI:** 10.3389/fpsyt.2014.00031

**Published:** 2014-03-27

**Authors:** Cecilie Skule, Hilde Dallavara Lending, Pål Ulleberg, Torkil Berge, Jens Egeland, Nils Inge Landrø

**Affiliations:** ^1^Community Mental Health Center Vinderen, Diakonhjemmet Hospital, Oslo, Norway; ^2^Department of Psychology, University of Oslo, Oslo, Norway; ^3^Psychiatric Department, Hospital of Vestfold, Tønsberg, Norway; ^4^Department of Psychology, University of Oslo, Oslo, Norway; ^5^Clinical Neuroscience Research Group, Department of Psychology, University of Oslo, Oslo, Norway

**Keywords:** alcohol, depression, coping, Beck depression inventory, prevention and control

## Abstract

Treatment-seeking patients (*N* = 233) were recruited as they started a course of relapse prevention and coping with depression. The mean Beck depression inventory (BDI-II) score was 26 points, indicating a moderate degree of depression. The sample was recruited from different outpatient clinics and screened for alcohol-related problems with the alcohol use disorders identification test (AUDIT). Almost half of the total sample had a score on AUDIT >8 indicating an alcohol problem. The participants in this study did not undergo a clinical interview to check out if their symptoms, as assessed with BDI-II and AUDIT, were part of a formal diagnosis in accordance with the criteria in ICD 10 or DSM IV. A specific instrument, perceived uncontrollability of depression (UNCONTROL), was used to measure the persons’ perceived control of depressive symptoms; a set of statements about coping with depressive symptoms where high scores indicate lack of coping with the symptoms. Alcohol problems were not found to be significantly associated with the perceived control of ongoing depressive symptoms and did not moderate the relationship between depressive symptoms and the perceived control of depressive symptoms. The results question the assumption that alcohol use is related to coping with depressive symptoms in patients with alcohol abuse and depressive symptoms.

## Introduction

Depression is often comorbid with alcohol-related problems (1–3). Among patients with a major depressive disorder around 30% will have a substance use disorder (4). Conversely, for patients seeking treatment for alcohol-related problems, depressive symptoms are also often present (5). The National Comorbidity Study found a high 12-month prevalence of both anxiety disorders (36.9%) and affective disorders (29.2%) in alcohol-dependent patients (6).

Although there is consistent evidence of an association between alcohol- and substance-dependence and depression (7), it is difficult to establish exact relations (8, 9). Clinicians often assume that patients use alcohol as an attempt to alleviate symptoms of depression (10–12), implicating reduced control of depressive symptoms. An erroneous assumption about the role of alcohol in mastering depressive symptoms may give a direction in treatment that is not helpful for the patient. However, evidence from studies to confirm or deny such assumptions is lacking.

The main aim of this study was to explore the assumption that alcohol use influences the perceived control of depression. Secondly, we investigated the role of alcohol as a possible moderator between the degree of depressive symptoms and perceived control of depression.

## Materials and Methods

### Participants

The sample consisted of 233 patients seeking help for depressive symptoms in the mental health care system. The proportion of the sample with a comorbid alcohol abuse problem was 46% (107 patients). The participants attended a group-based cognitive–behavioral treatment addressing depressive symptoms. Participants in the project did not have psychotic symptoms or acute suicidal symptoms. Most of the patients were recruited from Community Mental Health Centers. A small group was recruited from a substance abuse clinic.

### Instruments

### The Beck depression inventory – second edition

The Beck depression inventory – second edition [BDI-II (13)] is one of the most commonly used self-report instruments to estimate the severity of a depression. The total score gives an indication of a mild, moderate, or major depression. BDI-II consists of 21 items. Every item has four answering alternatives and is scored from 0 to 3. The maximum score is 63. The following values are recommended: total score 0–13 minimal depression, total score 14–19 mild, total score 20–28 moderate, and total score 29–63 refers to major depression. In the current sample, the mean score on depression was 26.1 (SD = 10.2). The distribution within the minimal, mild, moderate, and major depression categories was 9% (*n * = 22), 17% (*n * = 40), 34% (*n * = 79), and 40% (*n* = 92), respectively.

### Alcohol use disorders identification test

Alcohol use disorders identification test [AUDIT (14)]. The test consists of 10 items and can be administered as an interview or self-administered by the patient. It is developed by the World Health Organization. Each item is scored on a four-point scale ranging from 0 to 40, where total scores of 8 or more are recommended as indicators of harmful alcohol use, as well as possible alcohol dependence. The maximum score is 40. Based on research (14), the following categories have been identified: total score 0–7 low risk, 8–15 medium risk, 16–40 high risk. In the current sample, the mean score on AUDIT was 10.2 (SD = 9.0). The distribution within the three categories low, medium, and high risk was 54% (*n * = 126), 18% (*n * = 41), and 28% (*n * = 66), respectively.

### Perceived uncontrollability of depression

Perceived uncontrollability of depression [UNCONTROL (15)]. The instrument consists of 10 statements that indicate the degree of perceived control of depressive symptoms. In the current sample, the mean score on UNCONTROL was 40.1 (SD = 11.6). The reliability of the summated score of the 10 items estimated by Cronbach’s alpha was 0.894.

### Procedure

The project was approved by Regional Committees for Medical and Health Research Ethics. In addition to screening depressive symptoms, substance abuse, and perceived uncontrollability of depression, the participants answered questions about demographic issues and their experience with depression earlier in life. The patients completed the screening before the cognitive–behavioral treatment of depressive symptoms started. The research was funded by the Regional Competence Centre for Double Diagnoses, South-Eastern Norway and the research fund in the Community Mental Health Center, Vinderen, Diakonhjemmet Hospital, Oslo. Permanent external scientific guidance of the project ensured scientific independence in the interpretation of the data and findings.

## Results

Initial ANOVAs found no statistically significant differences between the three AUDIT-categories in mean scores on either level of depressive symptoms [*F*(2, 230) = 0.41, *p* = 0.75] or control of depression [*F*(2, 230) = 1.08, *p* = 0.34].

Hierarchical regression analysis was used to examine the effect of level of depressive symptoms and alcohol use upon control of depression (Table 1). In step 1, level of depressive symptoms and alcohol use were entered as predictors. The results showed a statistically significant relationship between level of depressive symptoms and control of depression; the higher the level of depressive symptoms, the less control of depression was reported (β = −0.46). Alcohol use was not found to be related to control of depression. In step 2, the possible moderator effect of alcohol use was tested by adding an interaction term between BDI-score and AUDIT-score in the regression model. No evidence for a moderator effect was found, as the inclusion of the interaction term did not increase the model’s fit to the data significantly (Δ*R*^2^ = 0.001, *p * = 0.56).

**Table 1:** **Hierarchical regression analysis**.

	*b*	(SE)	Beta	*R*^2^	Δ*R*^2^	*r* (zero-order)
**MODEL 1**						
BDI	−0.53	(0.07)	−0.46***			−0.46***
AUDIT	0.01	(0.08)	−0.01	0.214***		0.02
**MODEL 2**						
BDI	−0.49	(0.10)	−0.42***			
AUDIT	0.13	(0.22)	0.08			
BDI × AUDIT	−0.01	(0.01)	−0.11	0.215***	0.001	

**N* = 233*.

***p < 0.001.

*Effects of BDI-score and AUDIT-score on control of depression*.

Additional analyses tested possible main effects of gender and age upon control of depression, as well as possible moderator effects of gender and age upon the relationship between alcohol use and control of depression. No statistically significant main or moderator effects of these variables were found, and the results from these analyses are thus not reported. A possible curvilinear effect of alcohol use upon control of depression was also tested. No evidence for such an effect was found.

## Discussion

Alcohol use in patients with depressive symptoms did not influence the perceived control of depression, independent of the severity of depressive symptoms, or the degree of alcohol problems. Furthermore, we did not find a moderating effect of alcohol on the relation between BDI-II score and the perceived control of depression (UNCONTROL). As expected, a significant negative association between degree of depressive symptoms and perceived controllability was found.

Controversy about how to treat patients with concurrent disorders persists. One of the most basic questions is whether to treat depression in the setting of ongoing substance abuse. Clinicians working with substance-dependent patients are often reluctant to initiate specific antidepressant treatment due to concerns about confusing substance-induced depressive symptoms with true depressive disorders or that a focus on depression could distract attention from treatment of the addiction (16, 17). At the same time, it is often recommended that concurrent substance abuse should not be a barrier to treatment of depression (18) and that the addition of antidepressants or cognitive–behavioral therapy for depression is effective for treating alcohol- or substance-dependent patients with depression (19, 20). The clinical implications of our findings might be that patients with this comorbidity are in need of treatment addressing both depressive symptoms and alcohol problems. A biased assumption consisting of a causal relation between alcohol use and depressive symptoms can lead to a rigid clinical approach and represent a barrier toward a successful treatment.

A thinking style characterized by a less flexible and less reflected way of thinking has been identified in patients with early relapse to depression (15). Patients with this thinking bias may have problems formulating a nuanced description of their perceived control of depression. This topic should be investigated in future studies. The strength of this study is the relatively large clinical sample consisting of patients with depressive symptoms with and without comorbid alcohol problems recruited from the same area and in the same period.

There are some limitations in our study. The participants in this study did not undergo a clinical interview to check out if their symptoms, as assessed with BDI-II and AUDIT, were part of a formal diagnosis in accordance with the criteria in ICD 10 or DSM IV. Further, the patients in this study were patients seeking treatment for depressive problems, some of them with comorbid alcohol problems and some of them without comorbid alcohol problems. The results cannot be generalized to patients seeking help for alcohol use disorders symptoms, with or without depression, unless an assessment of the overall situation is performed.

Alcohol use does not play a significant role in patients’ perceived control of depressive symptoms. Alcohol use was not identified as a moderator between the degree of depressive symptoms and perceived control of such symptoms.

## Conflict of Interest Statement

The authors declare that the research was conducted in the absence of any commercial or financial relationships that could be construed as a potential conflict of interest.
